# Atypical age distribution and high disease severity in children with RSV infections during two irregular epidemic seasons throughout the COVID-19 pandemic, Germany, 2021 to 2023

**DOI:** 10.2807/1560-7917.ES.2024.29.13.2300465

**Published:** 2024-03-28

**Authors:** Wei Cai, Sophie Köndgen, Kristin Tolksdorf, Ralf Dürrwald, Ekkehard Schuler, Barbara Biere, Brunhilde Schweiger, Luise Goerlitz, Walter Haas, Thorsten Wolff, Silke Buda, Janine Reiche

**Affiliations:** 1 Unit 36, Respiratory Infections, Department of Infectious Disease Epidemiology, Robert Koch Institute, Berlin, Germany; 2 Unit 17, Influenza and Other Respiratory Viruses, Department of Infectious Diseases, National Influenza Centre, Robert Koch Institute, Berlin, Germany; 3 Unit 17, Influenza and Other Respiratory Viruses, Department of Infectious Diseases, Consultant Laboratory for RSV, PIV and HMPV, Robert Koch Institute, Berlin, Germany; 4 HELIOS KLINIKEN GmbH, Berlin, Germany

**Keywords:** respiratory syncytial virus, surveillance, genotype, lineage, phylogeny, acute respiratory infection, disease severity, children

## Abstract

**Background:**

Non-pharmaceutical interventions (NPIs) during the COVID-19 pandemic affected respiratory syncytial virus (RSV) circulation worldwide.

**Aim:**

To describe, for children aged < 5 years, the 2021 and 2022/23 RSV seasons in Germany.

**Methods:**

Through data and 16,754 specimens from outpatient sentinel surveillance, we investigated RSV seasonality, circulating lineages, and affected children’s age distributions in 2021 and 2022/23. Available information about disease severity from hospital surveillance was analysed for patients with RSV-specific diagnosis codes (n = 13,104). Differences between RSV seasons were assessed by chi-squared test and age distributions trends by Mann–Kendall test.

**Results:**

RSV seasonality was irregular in 2021 (weeks 35–50) and 2022/23 (weeks 41–3) compared to pre-COVID-19 2011/12–2019/20 seasons (median weeks 51–12). RSV positivity rates (RSV-PR) were higher in 2021 (40% (522/1,291); p < 0.001) and 2022/23 (30% (299/990); p = 0.005) than in prior seasons (26% (1,430/5,511)). Known globally circulating RSV-A (lineages GA2.3.5 and GA2.3.6b) and RSV-B (lineage GB5.0.5a) strains, respectively, dominated in 2021 and 2022/23. In 2021, RSV-PRs were similar in 1 – < 2, 2 – < 3, 3 – < 4, and 4 – < 5-year-olds. RSV hospitalisation incidence in 2021 (1,114/100,000, p < 0.001) and in 2022/23 (1,034/100,000, p < 0.001) was approximately double that of previous seasons’ average (2014/15–2019/20: 584/100,000). In 2022/23, proportions of RSV patients admitted to intensive care units rose (8.5% (206/2,413)) relative to pre-COVID-19 seasons (6.8% (551/8,114); p = 0.004), as did those needing ventilator support (6.1% (146/2,413) vs 3.8% (310/8,114); p < 0.001).

**Conclusions:**

High RSV-infection risk in 2–4-year-olds in 2021 and increased disease severity in 2022/23 possibly result from lower baseline population immunity, after NPIs diminished exposure to RSV.

**Key public health message**

**What did you want to address in this study and why?**

Respiratory syncytial virus (RSV) is globally a leading cause of acute lower respiratory tract infections in young children. Non-pharmaceutical interventions during the COVID-19 pandemic strongly impacted RSV circulation. We aimed to investigate two extraordinary consecutive RSV seasons during the pandemic in Germany, by characterising their timing, the RSV genetic lineages circulating, the affected ages among < 5-year-olds, and disease severity.

**What have we learnt from this study?**

The 2021 and 2022/23 RSV seasons, which occurred during the pandemic, started earlier than pre-COVID-19 RSV seasons, with a respective intense circulation of first RSV-A and then RSV-B. RSV-A and -B strains belonged to known globally circulating lineages. In 2021, 2–4-year-olds were at high RSV infection risk, while in the 2022/23 season, RSV disease severity increased, with higher proportions of children needing intensive care treatment and ventilation.

**What are the implications of your findings for public health?**

The atypical character of RSV seasons in 2021 and 2022/23 was possibly due to reduced baseline population immunity to RSV, after NPIs diminished their exposure to RSV. Prospective, year-round and age-stratified surveillance systems in primary and secondary care are of crucial importance to estimate the impact of RSV disease and to characterise circulating virus strains to enable timely healthcare delivery and prevention measures.

## Introduction

Respiratory syncytial virus (RSV) is a common respiratory pathogen that affects people at all ages worldwide. Especially in children < 5 years of age and older adults aged ≥ 65 years, RSV can cause acute lower respiratory infections (ALRI) such as bronchiolitis, pneumonia, or bronchitis. Within the first year of life, nearly 70% and by the end of the second year 90% of children have experienced an RSV infection at least once [1]. In 2019, 6.6 million and 5.6 million RSV-associated ALRI episodes were estimated to occur in infants aged < 6 months and aged 6–12 months worldwide, including 1.4 million and 0.7 million RSV-associated ALRI hospital admissions, respectively [2]. For children aged < 5 years, 33.0 million RSV-associated ALRI episodes globally were estimated, including 3.6 million RSV-associated ALRI hospital admissions [2].

Passive immunisation with monoclonal antibody palivizumab has been available for infants with a high-risk profile for more than 20 years in Europe and was authorised in Germany in 1999. In 2022, a novel monoclonal antibody (nirsevimab) with longer half-life, and in 2023 two vaccines (Arexvy and Abrysvo) against RSV were approved by the European Medicines Agency (EMA) [3]. Nirsevimab, Arexvy, and Abrysvo have been available in Germany since autumn 2023; however, recommendations for their administration are currently pending.

Based on antigenic and genetic variability RSV is subtyped into subgroups A and B. Viral strains of both subgroups circulate simultaneously. However, RSV-A dominates in most RSV seasons [4]. Moreover, within each subgroup, RSV are assigned to several genotypes and/or lineages, and recently, attempts have been made to define and propose criteria to bring uniformity to strain designations and thereby to description of virus evolution [5-9].

In European countries, RSV has a clear seasonality, with annual epidemics occurring during the winter months [10]. Focusing on Germany, which has a year-round surveillance for this virus, RSV circulates regularly from December to March with peaks in February [11]. In December 2019, the severe acute respiratory syndrome coronavirus 2 (SARS-CoV-2) emerged and spread rapidly around the world [12]. The World Health Organization subsequently declared the COVID-19 outbreak a global pandemic on 11 March 2020 [12]. Like in many other countries, several nonpharmaceutical interventions (NPIs) were implemented in Germany to control the spread of SARS-CoV-2 [13,14]. These included mandates to wear masks, physical distancing (≥ 1.5 m between people), and hygiene measures as a minimum. Over a period of 2 years, repeated contact restrictions of varying stringency were imposed, such as cancellation of mass gatherings (April–May 2020, June 2020, November 2020–April 2021), closures of schools and childcare facilities (March–July 2020, December 2020–May 2021) or complete closing of all nonessential shops (March 2020, December 2020–March 2021) [13]. Early analyses by sentinel surveillance showed that NPIs also precluded the transmission of many other respiratory viruses including RSV in Germany [14].

Here we report on two RSV seasons during the COVID-19 pandemic, which had an exceptional temporal occurrence, and describe the circulating RSV subgroups and lineages during these seasons, as well as the age characteristics of RSV cases aged < 5 years and their disease severity.

## Methods

### Data sources

For children aged < 5 years, comprehensive data analyses were performed in three settings, two in primary healthcare and one in secondary healthcare (Table 1). In primary healthcare, the surveillance was conducted by the German Influenza Working Group at the Robert Koch Institute (RKI) [15]. The two pillars of the primary care surveillance were on one hand, the virological surveillance, which informs on RSV since 2011 and on the other, the syndromic surveillance, which provides data since 2012. The syndromic surveillance relied on the sentinel electronic data collection system for patients with ARI (SEED^ARE^) based on International Statistical Classification of Diseases and Related Health Problems (ICD)-10 codes [16]. In secondary care, similar to SEED^ARE^, electronical medical data were gathered, but focused instead on hospitalised patients with severe ARI (SARI) through the ICD-10-code-based surveillance system for SARI (ICOSARI) [17]. The ICOSARI surveillance, established by the RKI in cooperation with a private hospital network in Germany, has been in place since 2015. Historical data from 2013 to 2014 were available for retrospective analysis.

**Table 1:** Characteristics of primary and secondary care sentinel surveillance systems that the Robert Koch Institute uses to monitor (severe) acute respiratory infections, Germany, 2011–2023

Surveillance type	Sentinel partners and sentinel distribution nationwide	Cases monitored by the surveillance and their definitions	Sample and/or data collection
Primary healthcare			
Virological surveillance [15]; historical RSV data since 2011	Approximately 115 medical practices (involving paediatricians, general practitioners and internists)^a^, which are evenly distributed over Germany and from all 16 federal states; these practices cover approximately 0.2% of the German population.	**Cases monitored in the 2011/12–2014/15 season:** *ILI cases* Any person with a sudden onset of symptoms AND fever or feverishness AND at least one of the respiratory symptoms cough or sore throat. *ARI cases* Any person with at least one of the following respiratory symptoms fever, cough, coryza or sore throat. **Cases monitored in the 2015/16–2020/21:** *ILI cases* Any person with a sudden onset of symptoms AND at least one of the following three systemic symptoms fever or feverishness, headache, myalgia AND at least one of the two following respiratory symptoms cough or sore throat. *ARI cases* Any person with at least one of the following respiratory symptoms fever, cough, coryza or sore throat**.** **Cases monitored in the 2021/22–2022/23:** *ARI cases only* Any person with at least one of the following respiratory symptoms fever, cough, coryza or sore throat.	**Sample collection for the seasons 2011/12–2022/23^b^** Sentinel practitioners were asked to randomly collect, on a year-round voluntary basis, one nasal and/or throat swab per week from each of the following age groups of ILI/ARI patients: 0 – ≤ 23 months AND ≥ 2 years – < 5 years and to send it to the RKI for laboratory testing for a panel of respiratory viruses, including RSV. **Data collection for the seasons 2011/12–2022/23** For each patient sampled, a standardised sample submission form was completed and sent along with the specimens. The form includes medical consultation data such as age, sex^c^, region, date of sampling, vaccination status (e.g. influenza, COVID-19), chronic or underlying diseases and symptoms.
Sentinel electronic data collection system for ARI based on ICD-10 codes (SEED^ARE^) [17,38,39]; historical data since 2012^d^	Approximately 500 medical practices (involving paediatricians, general practitioners and internists), which are evenly distributed over Germany, and from all 16 federal states; these practices cover 1% of German population.	***ARI case*** is defined as a patient with one of the ARI ICD-10 code diagnoses: J00–J22, J44.0, and B34.9. ***RSV case*** is defined as an ARI case with one of the three RSV-specific ICD-10 code diagnoses J12.1 (RSV pneumonia), J20.5 (acute bronchitis due to RSV), and J21.0 (acute bronchiolitis due to RSV).	On a year-round voluntary basis, sentinel practitioners sent weekly anonymised data on age, sex^c^, region, ICD-10 code diagnoses, date of consultation, information on incapacity to work, hospitalisation and vaccination status (e.g. influenza, COVID-19) of patients with any of the ARI ICD-10 code diagnoses (J00**–**J22, J44.0, B34.9) to the RKI.
Secondary healthcare			
ICD-10 based hospital surveillance for severe acute respiratory infections (ICOSARI) [17,39]; historical data since 2013	Approximately 70 hospitals, located in 13 federal states of Germany and covering to 5–6% of all hospitalised patients in Germany.	***SARI case*** is defined as a hospitalised case with one of the ALRI ICD-10 code diagnoses (J09–J22) as primary or secondary discharge diagnosis [39]. ***RSV case*** is defined as a SARI case with one of the three RSV-specific ICD-10 codes (J12.1, J20.5, J21.0, see above) as primary or secondary discharge diagnosis.	On a year-round voluntary basis, data on age, sex^c^, region, primary and secondary discharge ICD-10 code diagnosis, admission diagnosis, admission date, discharge date, length of stay in hospital and in intensive care unit, duration of ventilatory support and outcome of hospitalised patients with any of the respiratory ICD-10 codes (chapter X in [16]: J00–J99) as primary or secondary discharge diagnosis were sent to the RKI weekly.

ALRI: acute lower respiratory infection; ARI: acute respiratory infection; ICD-10 code:  International Statistical Classification of Diseases and Related Health Problems [16]; ICOSARI: ICD-10-code-based hospital surveillance for severe acute respiratory infections; ILI: influenza-like illness; RKI: Robert Koch Institute; RSV: respiratory syncytial virus; SARI: severe acute respiratory infection; SEED^ARE^: Sentinel electronic data collection system for ARI based on ICD-10 codes.

^a^ These practices resemble a subset of the SEED^ARE^ participating sentinel practices. Additional sentinel practices were recruited starting with the 2021/22 season. During the COVID-19 pandemic, 144 and 121 practices participated in the virological sentinel in the 2021/22 and 2022/23 seasons, respectively.

^b^ In order to maintain and/or to increase the number of specimens collected during the COVID-19 pandemic (2021/22–2022/23), sentinel practitioners were regularly contacted by study nurses. For children aged < 5 years, a median of 944 (range: 535–1,348) samples were collected for the seasons 2011/12–2019/20 and a median of 2,422 (range: 2,165–2,911) samples were collected for the seasons 2021/22–2022/23 during the COVID-19 pandemic.

^c^ Sex recorded at the time of medical consultation (male/female/unknown or missing data).

^d^ The RSV season in 2011/12 began in January 2012; therefore, the RSV data for the 2011/12 season were only available for the year 2012.

### Laboratory investigations

Respiratory specimens from outpatients participating in the virological sentinel surveillance were sent to RKI and prospectively screened by real-time PCR for a panel of respiratory viruses including RSV [14]. RSV-positive specimens were subtyped into RSV-A and RSV-B by real-time PCR (Supplementary Material). For the seasons 2018/19 to 2022/23, a subset of > 10% of RSV-A and -B positive specimens from children < 5 years of age with quantification cycle (Cq) ≤ 31 was randomly selected for in-solution hybridisation capture-based next generation sequencing. Consensus sequences were used for maximum-likelihood tree analysis of RSV-A and RSV-B, respectively [18]. Lineages were assigned according to Goya et al. [5]. Origin of RSV reference sequences used for the phylogenetic analyses are listed in the Supplementary Material (GISAID Acknowledgement RSV-A/RSV-B).

### Data analyses

Using virological surveillance, the start of an RSV season was defined as the first of 2 consecutive weeks in which the lower limit of the 95% confidence interval (CI) of the RSV positivity rate (PR) in children < 5 years of age exceeded 5%. The RSV season ended with the week preceding the first of 2 consecutive weeks in which the lower limit of the 95% CI of the RSV PR fell below 5% [11].

We calculated seasonal RSV PR (virological surveillance) and seasonal proportion of RSV specific diagnoses (J12.1, J20.5, J21.0) in ARI (SEED^ARE^) and in SARI cases (ICOSARI) with 95% CI for children < 5 years old and by age group (< 3 months; 3 – ≤ 5 months; 6 – ≤ 11 months, 12 months – < 2 years, 2 – < 3 years, 3 – <  4 years, 4 – < 5 years) in RSV seasons before and during the COVID-19 pandemic. Based on ICOSARI data, we further described characteristics of hospitalised RSV cases. Cumulative hospitalisation incidence was calculated as number of newly hospitalised RSV cases in an RSV season per 100,000 population [19]. Data on intensive care unit (ICU) admission and application of ventilator support of hospitalised RSV cases, respectively, were used for determination of RSV disease severity.

Mann–Kendall test was used to analyse trends in age distribution, chi-squared test to compare RSV PR, cumulative RSV hospitalisation incidence, proportion of RSV diagnoses, ICU admission, ventilation and death, between RSV seasons before and during the COVID-19 pandemic, and Mann–Whitney U test to compare length of hospitalisation, ICU stay and ventilation between RSV seasons before and during the COVID-19 pandemic. Non-overlapping 95% CI or a p value of < 0.05 was considered statistically significant. Stata (version 17) was used for data analyses.

## Results

### Irregular seasonal RSV activity during COVID-19 pandemic, 2020–2023

**Figure 1:**
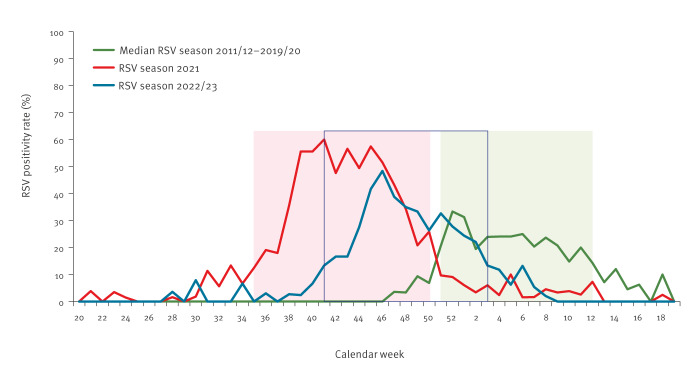
Respiratory syncytial virus weekly positivity rate for children aged < 5 years by calendar week, Germany, 2011–2023 (n = 16,754)

Using virological surveillance data from children aged < 5 years, RSV seasonality before and during the COVID-19 pandemic was compared (Figure 1). In pre-COVID-19 RSV seasons (2011/12–2019/20), a season started in median in calendar week 51 (range: 45–3), peaked in median in calendar week 8 (range: 51–10), and ended in week 12 (range: 10–18), and had a median length of 15 weeks (range: 13–18) [11].

RSV: respiratory syncytial virus.

Virological sentinel surveillance data for the 2021 and 2022/23 RSV seasons are compared with the median of the pre-COVID-19 RSV seasons 2011/12–2019/20. Boxes indicate the period of the seasons: median of 2011/12–2019/20 in green, 2021 in red, and 2022/23 in blue (frame only).

During the first pandemic winter in 2020/21, a regular RSV season was missing in Germany. Sporadic RSV cases occurred between weeks 9 and 30 in 2021. Following easing of NPI measures from May 2021 onwards [13], the number of RSV cases and corresponding RSV PR rapidly increased from week 31 onwards. The RSV season began in week 35 (PR: 13%; 95% CI: 5.2–24.1), peaked in week 41 (PR: 60%; 95% CI: 47.6–71.5), and continued until week 50 in 2021 (PR: 26%; 95% CI: 15.3–39.0) with a length of 16 weeks (Figure 1). Compared to previous seasonal patterns, the RSV circulation in 2021 resembled an unusually ‘inter-seasonal’ RSV activity and is therefore referred to as the ‘2021 season´ here. The subsequent 2022/23 RSV season started in week 41 in 2022 (PR: 13.3%; 95% CI: 5.1–26.8), peaked in week 46 in 2022 (PR: 48.4%; 95% CI: 37.7–58.5) and lasted for 15 weeks until week 3 in 2023 (PR: 13.3%; 95% CI: 5.9–24.6) (Figure 1). The 2021 season can be considered as a ‘late 2020/21 season’ or an ‘early 2021/22 season’; considering the latter, the 2021 and 2022/23 seasons started earlier than the pre-COVID-19 RSV seasons. The length of the seasons, however, remained the same.

### Intense RSV activity during the COVID-19 pandemic, 2020-2023

During the COVID-19 pandemic the RSV PR of both RSV seasons was significantly higher (2021: 40% (522/1,291); 95% CI: 38–43, p < 0.001; 2022/23: 30% (299/990); 95% CI: 27–33, p = 0.005) compared with pre-COVID-19 RSV seasons (2011/12–2019/20: 26% (1,430/5,511); 95% CI: 25–27) (Figure 1).

Based on SEED^ARE^ surveillance of ARI ICD10-code diagnoses in primary care, and RSV ICD10-code diagnoses, the proportion of RSV diagnoses among ARI cases in children aged < 5 years was significantly higher in the 2021 (1.6% (824/51,509); 95% CI: 1.5–1.7; p < 0.001) and 2022/23 (1.4% (810/59,968); 95% CI: 1.3–1.4; p < 0.001) seasons than in pre-COVID-19 seasons (2011/12–2019/20: 0.5% (988/188,173); 95% CI: 0.5–0.6).

In secondary care (ICOSARI), for children aged < 5 years, the proportions of RSV diagnoses among SARI cases were ca twofold higher in 2021 (60.0% (2,577/4,295); 95% CI: 58.5–61.5, p < 0.001) and 2022/23 (52.1% (2,413/4,631); 95% CI: 50.7–53.6, p < 0.001) than in the pre-COVID-19 seasons (31.5% (8,114/25,757); 95% CI: 30.9–32.1) (Table 2). The respective cumulative incidences of RSV hospitalisation in the 2021 (1,114 per 100,000; 95% CI: 1,072–1,158, p < 0.001) and 2022/23 (1,034 per 100,000; 95% CI: 993–1,076, p < 0.001) RSV seasons were also about twofold higher than the average of the pre-COVID-19 2013/14–2019/20 RSV seasons (584/100,000; 95% CI: 554–616). 

**Table 2:** Characteristics of hospitalised RSV cases aged < 5 years by season, ICOSARI surveillance data, Germany, 2013–2023 (n = 13,104)

Variable	Pre-COVID-19 RSV seasons (2013/14–2019/20)	RSV season 2021	Comparison pre-COVID-19 and 2021 season p value	RSV season 2022/23	Comparison pre-COVID-19 and 2021/22 season p value
Number of hospitalised cases with RSV diagnosis	1,159^a^ (male 56%, female 44%)	2,577 (male 59%, female 41%)	NA	2,413 (male 57%, female 43%)	NA
Cumulative RSV hospitalisation incidence per 100,000	584^b^ (95% CI: 554–616)	1,114 (95% CI: 1,072–1,158)	< 0.001^c^	1,034 (95% CI: 993–1,076)	< 0.001^c^
Proportion of RSV diagnoses among SARI cases	31.5% (8,114/25,757) (95% CI: 30.9–32.1)	60.0% (2,577/4,295) (95% CI: 58.5–61.5)	< 0.001^c^	52.1% (2,413/4,631) (95% CI: 50.7–53.6)	< 0.001^c^
Proportion of ICU admission among hospitalised RSV cases	6.8% (551/8,114) (95% CI: 6.3–7.4)	5.2% (135/2,577) (95% CI: 4.4–6.2)	0.005^c^	8.5% (206/2,413) (95% CI: 7.5–9.7)	0.004^c^
Proportion of ventilation among hospitalised RSV cases	3.8% (310/8,114) (95% CI: 3.4–4.3)	3.8% (98/2,577) (95% CI: 3.1–4.6)	0.967^c^	6.1% (146/2,413) (95% CI: 5.1–7.1)	< 0.001^c^
Proportion of deaths among hospitalised RSV cases	0.06% (5/8,114) (95% CI: 0.03–0.1)	0	0.208^c^	0.08% (2/2,413) (95% CI: 0.01–0.3)	0.723^c^
Median length of hospital stay among hospitalised RSV cases	4 days (IQR: 3–6)	3 days (IQR: 2–5)	< 0.001^d^	4 days (IQR: 2–6)	< 0.001^d^
Median length of ICU stay among ICU admitted RSV cases	5 days (IQR: 3–8)	5 days (IQR: 3–7)	0.183^d^	4 days (IQR: 2–7)	0.005^d^
Median ventilation length among ventilated RSV cases	98 hours (IQR: 53–157)	100 hours (IQR: 60–128)	0.504^d^	96 hours (IQR: 57–144)	0.765^d^

ICU: intensive care unit; IQR: interquartile range; NA: not applicable; RSV: respiratory syncytial virus; SARI: severe acute respiratory infection; 95% CI: 95% confidence interval.

^a^ Mean of hospitalised RSV cases.

^b^ Mean cumulative RSV hospitalisation incidence only for RSV seasons 2014/15–2019/20.

^c^ Chi-squared test.

^d^ Mann–Whitney U test.

### Atypical age distribution in 2021 RSV season

**Figure 2:**
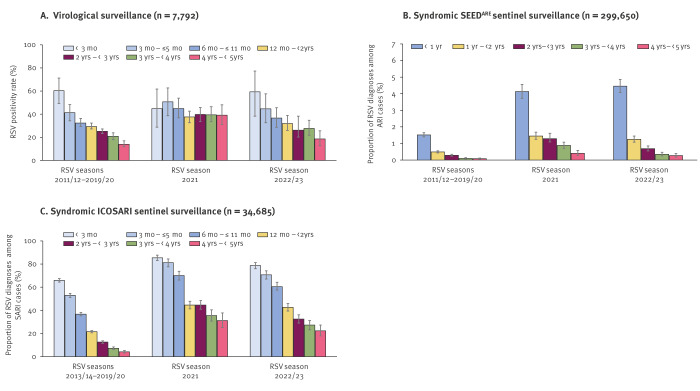
RSV positivity rate and proportion of RSV cases among ARI/SARI cases with 95% confidence interval by age group and RSV season, Germany, 2011–2023

The age distributions of RSV cases during the pandemic and in the pre-COVID-19 RSV seasons found in the primary healthcare virological and syndromic surveillance (SEED^ARE^) and the secondary healthcare ICOSARI are depicted in Figure 2.

ARI: acute respiratory infection; RSV: respiratory syncytial virus; SARI: severe acute respiratory infection.

In virological outpatient surveillance of children aged < 5 years, RSV PR significantly decreased with increasing age in the pre-COVID-19 RSV seasons (60–14%, p = 0.0027) and also in the 2022/23 season (59–19%, p = 0.0069) (Figure 2A). Interestingly, this pattern was not observed in the 2021 season (p = 0.13), where RSV PR was similarly high in children aged 1 – < 2, 2 – < 3, 3 – < 4, and 4 – < 5 years (38–40%) (Figure 2A).

Based on ARI ICD10-code diagnoses in primary care (SEED^ARE^), the proportion of RSV diagnoses decreased significantly with increasing age up to 5 years in RSV seasons before and during the COVID-19 pandemic (2021, 2022/23, 2011/12–2019/20: p = 0.0275) (Figure 2B). However, in the 2021 RSV season, the RSV proportion was similar in children aged 1 – < 2 year (1.5%; 95% CI: 1.3–1.7) and 2 – < 3 years (1.3%; 95% CI: 1.1–1.5).

In secondary care (ICOSARI), the proportion of RSV diagnoses decreased significantly with age in children aged < 5 years in all RSV seasons (2021: p = 0.0069; 2022/23: p = 0.0027; 2013/14–2019/20: p = 0.0027). Nevertheless, there were increases in the proportion of RSV diagnoses for 3- (2021: 4.8-fold, 2022/23: 3.7-fold) and 4-year-old children (2021: 7.2-fold, 2022/23: 5.3-fold) compared to the pre-pandemic seasons (Figure 2C). 

### Increased severity of RSV disease in the 2022/23 RSV season

Compared with pre-COVID-19 RSV seasons, in the 2021 season, the proportion of RSV cases admitted to the ICU among hospitalised RSV cases was reduced (p = 0.005), while the proportion needing ventilator support remained unchanged (p = 0.967; Table 2).

**Figure 3:**
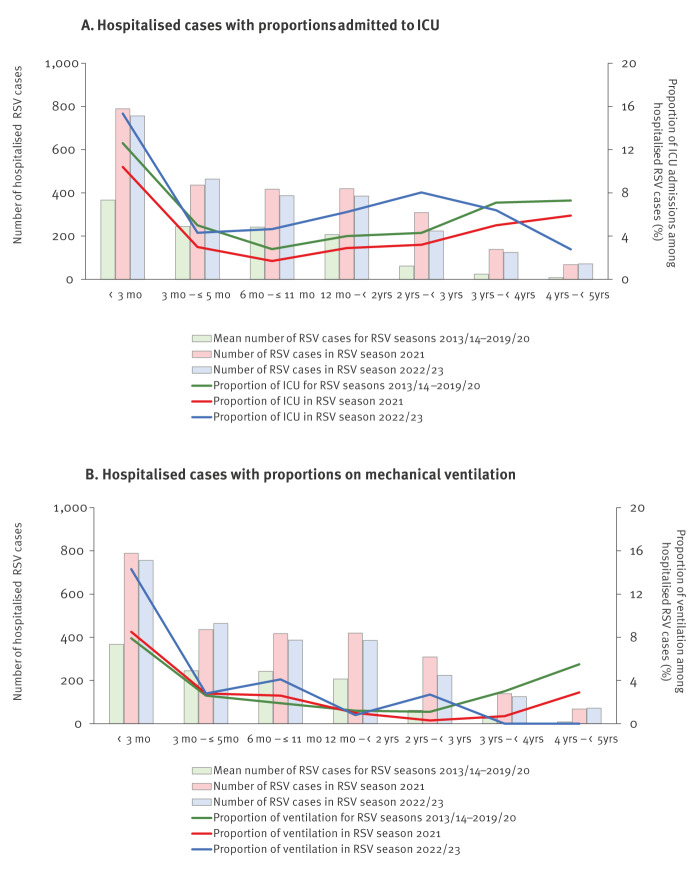
Number of hospitalised RSV cases and proportion of ICU admission or ventilation among hospitalised RSV cases by age and RSV season within ICOSARI surveillance, Germany, 2011–2023 (n = 13,106)

In the 2022/23 season, a significantly higher proportion of RSV cases aged < 5 years was admitted to ICU (p = 0.04) and required ventilator support (p < 0.001) compared with pre-COVID-19 RSV seasons. In particular, in infants aged < 3 months the proportion of ventilation was significantly increased (p < 0.001) (Figure 3). In addition, the proportions of ICU admission and ventilation were also increased in some other age groups, e.g. 2 − < 3 years old children, although not significantly. 

ICU: intensive care unit; No: number; RSV: respiratory syncytial virus.

Chi-squared test was used to compare the proportion of ICU admissions and ventilation among hospitalised RSV cases by age group between RSV seasons before and during the COVID-19 pandemic. No significant differences were found in ICU admission (p > 0.05). The proportion of children requiring ventilation was significantly higher in infants aged < 3 months in the 2022/23 season compared with pre-COVID-19 seasons (p < 0.001). No significant differences were found in the other age groups (p > 0.05).

### Occurrence of a monophyletic clade of RSV-B strains in the 2022/23 RSV season

**Figure 4:**
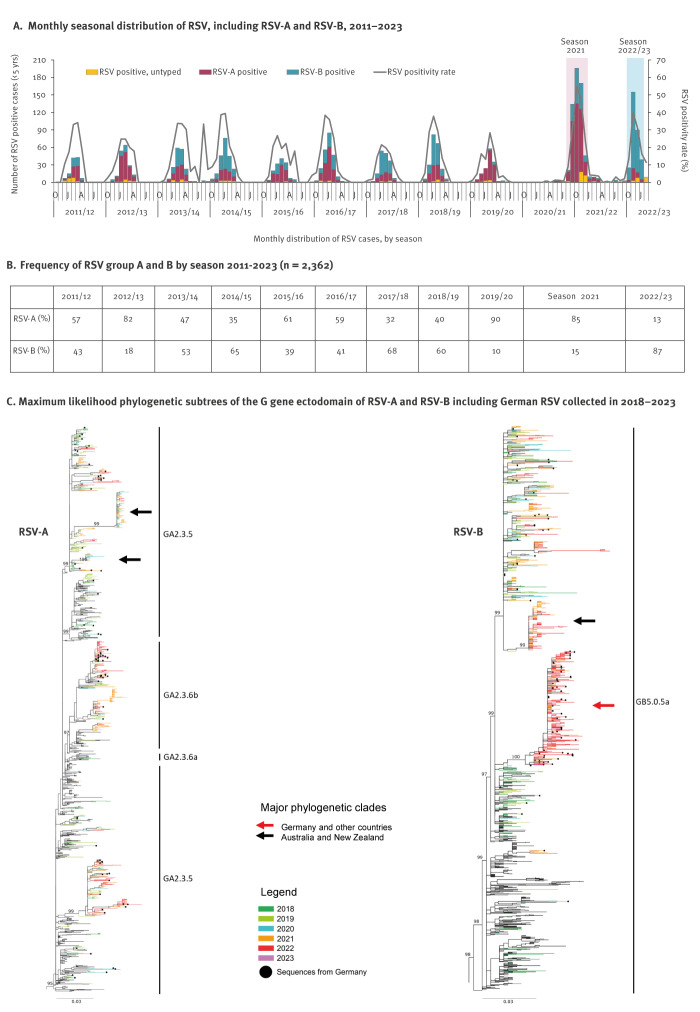
Seasonal distribution and phylogenetic characterisation of RSV-A and RSV-B, Germany, 2011–2023

Based on virological surveillance data, RSV subgroup A and B viruses co-circulated in each of the pre-COVID-19 seasons 2011/12 to 2019/20. However, RSV-A was dominant in five of nine seasons, up to an extent of 90% in season 2019/20 (Figure 4). Interestingly, the proportion of RSV-A remained unchanged in the 2021 season (85%), while RSV-B predominated in the most recent 2022/23 season (87%) (Figure 4).

RSV: respiratory syncytial virus; RSV-A: RSV subgroup A; RSV-B: RSV subgroup B.

A: RSV seasons 2021 and 2022/23 are highlighted by coloured boxes.

C: For the period 2018–2023, RSV sequences were coloured on their branches according to their collection year. German RSV sequences are additionally highlighted by black dots. Identical sequences collected in the same sampling location (country) and year or season, respectively, were removed and one representative left for phylogenetic analyses. The red arrow indicates a major monophyletic clade comprising RSV-B sequences from Germany and other countries in the world, collected almost exclusively during the COVID-19 pandemic in 2020–2023; black arrows indicate monophyletic clades of strains originating almost exclusively from Australia and New Zealand. Ultrafast bootstrap values are shown at major branches. Complete phylogenetic trees are provided in the Supplementary Figures S1 and S2.

Genome sequencing was performed for RSV-A and -B specimens collected between seasons 2018/19 and 2022/23. Phylogenetic analysis revealed that German RSV-A viruses belong to lineages GA2.3.5 and GA2.3.6b and RSV-B viruses to GB5.0.5a, respectively, and remained unchanged during this period of observation. While German RSV-A strains from the 2021 and 2022/23 seasons evolved in a manner typically observed for a continued season-to-season RSV circulation, the majority of recently circulating RSV-B strains were predominantly in a statistically supported monophyletic clade of GB5.05a (Figure 4). For viruses from Australia and New Zealand, monophyletic clades were identified for RSV-A and RSV-B, respectively; of note, the RSV monophyletic clade in these countries was not the same than the one in Germany.

## Discussion

In this study we reported on RSV circulation during the COVID-19 pandemic in Germany. Moreover, in children aged < 5 years, we analysed the age distribution of RSV cases and the RSV disease severity using data from our national primary and secondary care sentinel surveillance. While findings from similar investigations in other countries have been published, we also conducted phylogenetical analyses of the circulating RSV strains.

During the COVID-19 pandemic, we observed two irregular RSV seasons in Germany. Although these seasons started earlier, there was no difference in season length compared with pre-COVID-19 RSV seasons [10,11]. 

In the primary care setting of our study, RSV activity was significantly increased in children aged < 5 years in both the 2021 and 2022/23 seasons. For example, in the virological surveillance in 2021, there was an atypical age distribution with similarly high RSV PR in children aged 1, 2, 3, and 4 years. However, in SEED^ARE^, similar proportions of RSV diagnoses were only observed in children aged 1 and 2 years in 2021. Differences in the results of these two surveillance systems may be due to the fact that physicians in German primary healthcare settings rarely request diagnostic testing for suspected RSV infection, especially in toddlers aged 2–4 years, possibly because of financial constraints and the lack of effective therapeutic options. As a result, SEED^ARE^ physicians often diagnose suspected RSV cases using generic ARI ICD-10 codes instead of RSV-specific codes especially for individuals > 2 years-old. In addition, during the COVID-19 pandemic, sentinel practitioners participating in the virological surveillance were regularly contacted to maintain and/or to increase the number of specimens. Besides, when concerned about respiratory symptoms, patients might have had different behaviours during the COVID-19 pandemic and avoided visits to physicians unless absolutely necessary (e.g. obtaining sick notes subsequent to telephone or online consultations). Moreover, for ARI, centres for SARS-CoV-2 testing may have been prioritised over practices participating in the RSV virological and syndromic surveillance. While this may have influenced the RSV PR or proportion of RSV diagnoses in both primary care settings, results arising from either of these settings nevertheless suggest changes in the RSV-affected age groups among children < 5 years. Atypical age group distribution of RSV cases during the pandemic was also described by Casalegno et al. for 2020/21 in France [20]. For Germany, a possible explanation for the observed age shift could be the lack of RSV circulation in winter 2020/21 when children aged < 2 years did not have a first or booster RSV infection. Therefore, they may not have developed protective immunity, leading toddlers aged 2–4 years in the 2021 season to be more susceptible to RSV infection.

Similarly, in the secondary care setting of our study, the cumulative RSV hospitalisation incidence and the proportion of hospitalised RSV cases among SARI cases in children aged < 5 years were about twofold higher in the 2021 and 2022/23 RSV seasons in Germany compared to pre-COVID-19 seasons. The highest increase was observed in 3- and 4-year-old children. At least for 2020/21, other studies reported a similar increase in hospital admissions in children ≤ 5 years old, with the highest increase in children aged 2–5 years because of postponed primary RSV infection or waning immunity [20-22].

Interestingly, significantly fewer RSV cases were admitted to ICU in the 2021 season in our study, possibly because more children aged 2–4 years were affected compared with the 2022/23 and pre-COVID-19 seasons. These children are less likely to experience a severe infection because their immune system, airways, and respiratory muscle capacity are more developed than in infants [23,24]. During the first upsurge of RSV after relaxation of NPIs, reduced ICU admission was also reported from Australia in 2020 [25], whereas ICU admission increased in New Zealand and the United States (US) in 2021 [26,27]. At least in the US, the median age of the cohort was 6 months, making ICU admission more likely in these infants than in older children [27]. Furthermore, in the 2021 season, the proportion of German RSV cases receiving ventilator support was similar to pre-COVID-19 seasons. Similarly, in Danish children hospitalised for RSV, there was no difference in the risk of mechanical ventilation between RSV seasons before and during the COVID-19 pandemic [21].

Disease caused by RSV appears to be more severe in the 2022/23 season in Germany, with a significantly higher proportion of RSV cases being admitted to ICU and requiring ventilatory support compared with pre-COVID-19 seasons. This was particularly the case in infants aged < 3 months, who may be at high risk due to decreased levels of maternally derived antibodies following reduced RSV exposure in pregnant women [21,28]. Similarly, RSV contributed to more severe disease in children aged < 5 years during the 2022/23 RSV season in Colorado [29].

The increased number of RSV cases in both RSV seasons during the COVID-19 pandemic raises the question of whether the RSV strains of seasons 2021 and 2022/23 were genetically different from viruses circulating in pre-pandemic RSV seasons. In detail, RSV-A was detected in most RSV cases in the 2021 season, with viruses present in multiple clades within lineages GA2.3.5 and GA2.3.6b in our study and elsewhere, and lineages circulating globally for > 10 years [30-32]. In contrast, RSV-B, which last dominated in Germany in 2018/19 (60%), was the dominant subgroup in the 2022/23 season (87%). All RSV-B in this study belong to lineage GB5.0.5a, the current global circulating lineage [5,30-32]. Remarkably, the majority of German RSV-B from the 2022/23 season presented in a monophyletic clade with only RSV viruses collected since 2020, e.g. from the US [30,31], Argentina, South Africa, and other European countries. However, analyses of all viral genes from 2022/23 US RSV-A and -B showed no specific nonsynonymous changes compared with other RSV strains collected globally since 2017, suggesting that recent RSV variants alone are not responsible for increased viral spread during the COVID-19 pandemic [30]. In addition, two distinct monophyletic lineages within RSV-A were observed in Australia in 2020/21 [33]. Although both lineages were defined by several nonsynonymous mutations, implying selective pressure on virus evolution, the original ancestors, presumed to have emerged 1–2 years ago, could not be identified. Interestingly, a similar pattern was also observed for RSV-B in Australia, where the number of detections was very low [33]. As a monophyletic pattern of RSV-B strains circulating after 2020 was identified in Germany for the 2022/23 season, in line with Australia, this observation may reflect a bottleneck effect due to limited opportunities for RSV-B to circulate over such a long period, rather than the emergence of an altered virus strain [33,34]. Therefore, the increase in RSV cases in the 2021 and 2022/23 seasons in Germany is more likely to be associated with poorly developed or diminished immunity in the population [31,33]. Normally, maternal immunoglobulin G (IgG) concentrations decline by the age of 10–12 months, and antibody concentrations reach a plateau by the age of 5–9 years and remain constant throughout life [35]. However, a serological survey conducted between June 2020 and June 2021 in the Netherlands showed that RSV-specific IgG declined in all age groups when RSV circulation was limited or absent [36]. With regard to the children in our study, it can be assumed that protective immunity was not established as in previous years. Looking at the current 2023/24 RSV season in Germany, which began in week 47 in 2023 and is still ongoing, the timing seems to have returned to the usual pattern, while the number of RSV cases appears to remain at a similar level to that observed in RSV seasons during the COVID-19 pandemic [37].

The study’s main strength is the structured collection of population-based health data in primary and secondary care. A limitation of the study could be the recording of ICD-10 code diagnoses and their dependence on either clinician decision and/or the use of laboratory diagnostic tests in relation to assumptions on the RSV season timing and its epidemic situation or the age of the patients, thus over- or underestimating the real number of RSV cases. However, the use of ICD-10 codes has been proven valuable and useful for the assessment of RSV cases and is consistent with observations of surveillance systems in other countries [17].

### Conclusions

Overall, both RSV seasons had an earlier onset and greater intensity compared with pre-COVID-19 RSV seasons. In season 2021, there was a dominance of RSV-A, whereas in 2022/23 RSV-B was more prevalent. The RSV-A and -B strains identified each belong to known lineages that have circulated globally before and during the COVID-19 pandemic. Further, season 2021 showed an unusual age distribution of RSV cases with more cases in toddlers. In contrast, in season 2022/23, RSV disease was more severe, leading to more ICU admissions and cases needing mechanical ventilation. Therefore, year-round prospective primary and secondary care surveillance systems are of crucial importance to estimate the burden of RSV and thus enabling timely actions for healthcare service delivery and prevention.

## Supplementary Data

70000 Supplementary Material

## Supplementary Data

1143000 Supplementary Figure S1

## Supplementary Data

1280000 Supplementary Figure S2
