# Supplementary material for: Atypical age distribution and high disease severity in children with RSV infections during two irregular epidemic seasons throughout the COVID-19 pandemic, Germany, 2021 to 2023
Source: Euro Surveill. 2024 Mar 28;29(13):2300465. doi: 10.2807/1560-7917.ES.2024.29.13.2300465 (PMC10979527; doi:10.2807/1560-7917.ES.2024.29.13.2300465)
Supplement: Supplementary Material [file Supplementary_Material_2300465.pdf]

This supplementary material is hosted by Eurosurveillance as supporting information alongside the article

"Atypical age distribution and high disease severity in children with RSV infections during two irregular epidemic seasons throughout the COVID-19 pandemic, Germany, 2021-2023"

on behalf of the authors, who remain responsible for the accuracy and appropriateness of the content. The same standards for ethics, copyright, attributions and permissions as for the article apply. Supplements are not edited by Eurosurveillance and the journal is not responsible for the maintenance of any links or email addresses provided therein.

Supplementary Methods

Laboratory investigations

PCR-based RSV-A and -B subtyping was initially conducted in a two-step reaction chemistry using an oligonucleotide set modified from [1]. Reaction conditions are described in detail in [2]. Starting in March 2022, a one-step chemistry including the Luna® Probe One-Step RT-qPCR Kit (No ROX) (New England Biolabs) and a different oligonucleotide set (modified from [3]) was applied. In a 10µl reaction volume, 3µl of template underwent a cycling protocol comprising a 15 minutes reverse transcription and 45 cycles with annealing at 60°C. All oligonucleotides are listed in Supplementary Table 1.

Both PCR protocols target the RSV N-Gene and are based on a duplex qPCR principle. Differentiation of RSV subgroups A and B was achieved by labeling of probes with differing reporter dyes (6FAM/Yakima Yellow).

Synthetic duplex DNA molecules (GeneArt Strings, ThermoFisher Scientific) were used as reference material for both subgroups, each comprising the assay target region plus flanking sequences of the corresponding type. For the one-step protocol, the synthetic DNA also contained a T7 promotor sequence to allow for RNA synthesis applying the HiScribe® T7 High Yield RNA Synthesis Kit (New England Biolabs). After DNA digestion (Turbo DNase, ThermoFisher Scientific), synthesized RNAs were purified with the NucleoSpin RNA Clean-up kit (Macherey Nagel) and quantified with the Qubit™ RNA Broad Range Assay Kit (ThermoFisher Scientific) for copy number calculations.

Supplementary Table S1: Oligonucleotide sequences used for subtyping of RSV-A and RSV-B

Oligonucleotide	Sequence (5′ – 3′)	final concentration [nM]
two step protocol		
RSV-A F	AGATCAACTTCTRTCATCCAGCAA	500
RSV-A R	TTCTGCACATCATAATTAGGAGTRTCAAT	500
RSV-A TM	6FAM – ACCATCCAACGGAGCACAGGAGA – BHQ-1	100
RSV-B F	AAGATGCAAATCATAAATTCACAGGA	500
RSV-B R	ACATGATATCCAGCATCTTTAAGTATCTTTATA	500
RSV-B MGB	VIC – TTCCCTTCCTAACCTGGACA – MGB*	100
one step protocol		
RSV AB F	ARATGGCTCTTAGCAAAGTCAAGT	800
RSV AB R	TGCACATCATAATTRGGAGTGTCA	800
RSV AB TM-A	6FAM – CACTCAACAAAGATCAACTTCTATCATCCAGC– BHQ-1	200
RSV AB TM-B	YAK – CATTAAATAAGGATCAGCTGCTGTCATCCA– BHQ-1	200

BHQ-1: Black Hole Quencher 1 (dark quencher); MGB: Minor Groove Binder probe. TM: TaqMan probe; VIC: 2'-Chloro-7'-phenyl-1,4-dichloro-6-carboxyfluorescein (fluorescence dye); YAK: 5′-Yakima Yellow (fluorescence dye); 6FAM: 6-carboxyfluorescein (fluorescence dye)

References

1. van de Pol, A.C., et al., *Molecular quantification of respiratory syncytial virus in respiratory samples: reliable detection during the initial phase of infection.* J Clin Microbiol, 2010. **48**(10): p. 3569-74.
2. Oh, D.Y., et al., *Trends in respiratory virus circulation following COVID-19-targeted nonpharmaceutical interventions in Germany, January - September 2020: Analysis of national surveillance data.* Lancet Reg Health Eur, 2021. **6**: p. 100112.
3. Wang, L., et al., *Duplex real-time RT-PCR assay for detection and subgroup-specific identification of human respiratory syncytial virus.* J Virol Methods, 2019. **271**: p. 113676.
4. Goya, S., et al., *Toward unified molecular surveillance of RSV: A proposal for genotype definition.* Influenza Other Respir Viruses, 2020. **14**(3): p. 274-285.
