# Supplementary material for: Atypical age distribution and high disease severity in children with RSV infections during two irregular epidemic seasons throughout the COVID-19 pandemic, Germany, 2021 to 2023
Source: Euro Surveill. 2024 Mar 28;29(13):2300465. doi: 10.2807/1560-7917.ES.2024.29.13.2300465 (PMC10979527; doi:10.2807/1560-7917.ES.2024.29.13.2300465)
Supplement: Supplementary Figure S1 [file Supplementary_FigureS1_2300465.pdf]

This supplementary material is hosted by Eurosurveillance as supporting information alongside the article

“Atypical age distribution and high disease severity in children with RSV infections during two irregular epidemic seasons throughout the COVID-19 pandemic, Germany, 2021-2023”

on behalf of the authors, who remain responsible for the accuracy and appropriateness of the content. The same standards for ethics, copyright, attributions and permissions as for the article apply. Supplements are not edited by Eurosurveillance and the journal is not responsible for the maintenance of any links or email addresses provided therein.

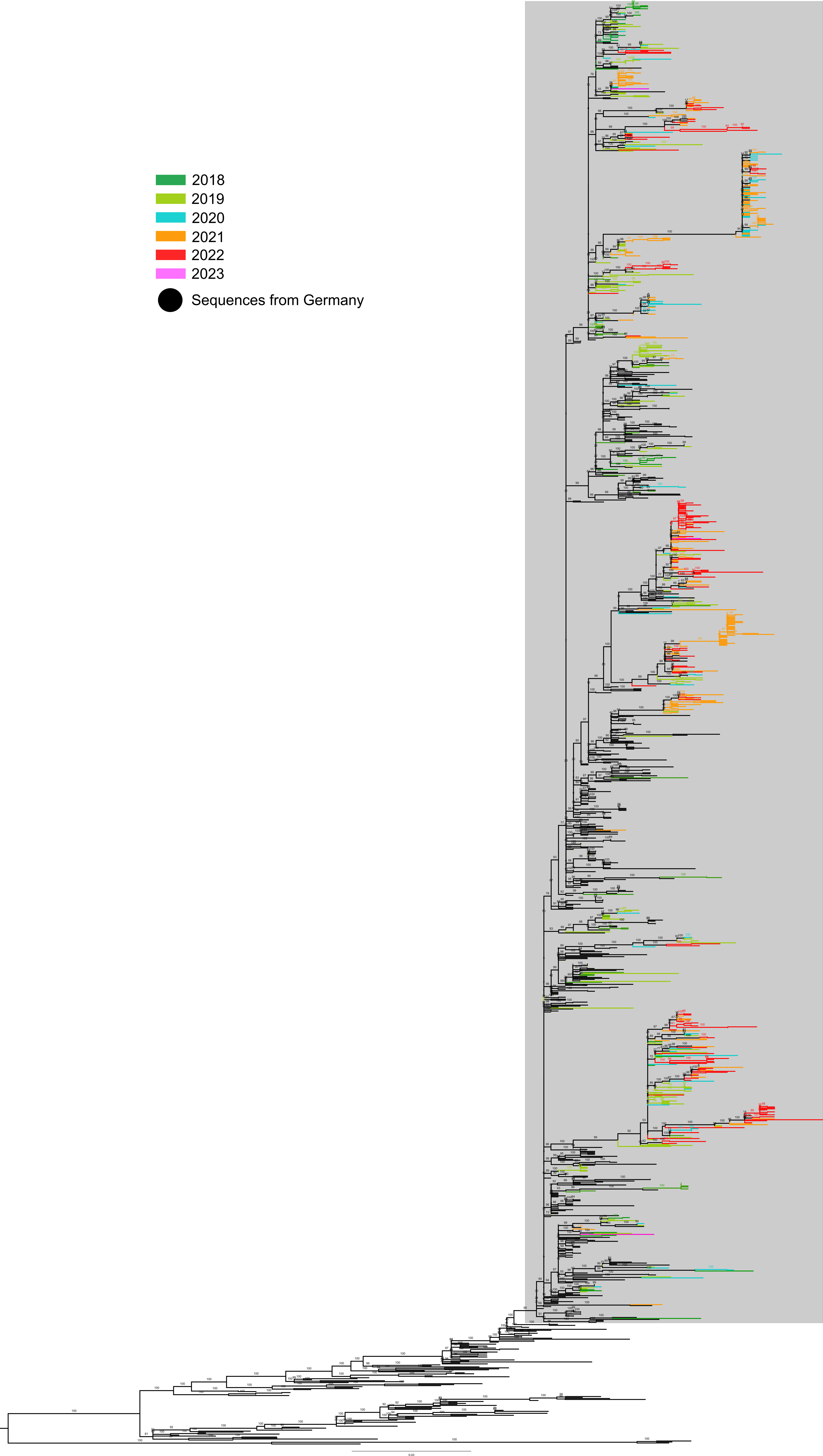
2018
2019
2020
2021
2022
2023
Sequences from Germany
0.03

Supplementary Figure S1. Maximum-likelihood phylogenetic tree analysis of the G gene ectodomain of RSV-A circulating in Germany, 2018-2023 (n= 100).

This tree marks the subtree (grey background) as presented in the main manuscript among reference sequences provided by Goya et al. [4]. Within subtree, German and other reference sequences collected between 2018 and 2023 were colored on their branches with respect to their collection year. Ultrafast bootstrap values are shown at major branches.
